# Enhancing risk stratification in diabetic gastric cancer: muscle-fat ratio from photon-counting CT as a predictor of postoperative complications

**DOI:** 10.1186/s12880-025-01872-1

**Published:** 2025-08-19

**Authors:** Shuangxiang Lin, Yuchen Jin, Mengxi Xu, Shuyue Wang, Weisheng Yao, Jiaxing Wu, Xinhong Wang, Jianzhong Sun

**Affiliations:** 1 Department of Radiology, The Second Affiliated Hospital Zhejiang, University School of Medicine, Hangzhou, 310000 China; 2 Department of Internal Medicine, Linhai Maternal and Child Health Care Hospital, Taizhou, 317000 China; 3 Siemens Healthineers, No. 399, West Haiyang Road, Shanghai, 200126 China

**Keywords:** Muscle-to‐fat ratio, Photon-counting CT, Diabetic, Gastric cancer

## Abstract

**Background:**

In diabetic gastric cancer patients, body composition (skeletal muscle–to–fat ratio, MFR) may influence surgical outcomes. We evaluated whether Photon-counting CT (PCD-CT) derived MFR predicts major postoperative complications, reflecting its value in perioperative risk stratification.

**Methods:**

A retrospective analysis of 134 gastric cancer patients with type 2 diabetes was conducted. Preoperative PCD-CT scans assessed body composition. Logistic regression models identified predictors of poor postoperative outcomes, defined by major postoperative complications. The predictive accuracy of models incorporating clinical variables and MFR was evaluated using receiver operating characteristic curves, integrated Discrimination Improvement (IDI), and net Reclassification Improvement (NRI).

**Results:**

Patients who developed major complications (*n* = 35) had significantly lower skeletal muscle area (45.5 vs. 56.2 cm²; *P* < 0.01) and higher fat accumulation. Abnormal MFR (0.34–0.57)was a strong predictor of poor outcomes (OR = 1.94, 95% CI: 1.17–2.58, *p* < 0.01) compared to patients without complications (*n* = 99). The model combining clinical variables with MFR had the best performance (AUC = 0.75, sensitivity = 0.74, specificity = 0.71) in predicting major complications, outperforming a model based solely on clinical factors. It also showed substantial improvements in predictive accuracy, with an NRI of 0.52 (*p* < 0.01) and an IDI of 0.09 (*p* < 0.01).

**Conclusion:**

MFR, quantified by PCD-CT, is a reliable and accurate biomarker for identifying diabetic gastric cancer patients at higher risk of major postoperative complications. MFR demonstrates strong predictive value for adverse surgical outcomes, reinforcing its role in perioperative risk stratification.

**Clinical trial number:**

Not applicable.

**Supplementary Information:**

The online version contains supplementary material available at 10.1186/s12880-025-01872-1.

## Introduction

Gastric cancer remains a major global health concern, with an estimated 1.09 million new cases and 768,000 deaths annually, ranking as the fourth most prevalent cancer and the second leading cause of cancer-related mortality worldwide [1, 2]. Emerging evidence highlights the multifactorial determinants of gastric cancer prognosis, including tumor stage, histological subtype, and patients’ metabolic status [3]. Among these factors, diabetes mellitus has been identified as a significant predictor of poorer outcomes in patients with gastric cancer. Notably, individuals with diabetes have a 24% higher risk of developing gastric cancer and tend to experience worse surgical outcomes, including increased 90-day postoperative mortality and a 55% greater likelihood of major complications [4, 5]. The complex interplay between diabetes and gastric cancer progression, mediated by metabolic dysregulation affecting both the tumor microenvironment and overall physiological resilience, underscores the urgent need for improved risk stratification tools tailored to this vulnerable population [6].

Body composition, particularly the muscle-fat ratio (MFR), has become a crucial prognostic indicator in cancer patients [7, 8]. Unlike traditional metrics such as BMI, the MFR has gained attention as a composite biomarker reflecting nutritional-metabolic equilibrium [9]. Despite its prognostic relevance in chronic diseases, two critical knowledge gaps hinder clinical application: lack of standardized diagnostic thresholds, particularly for metabolic disorders, and context-dependent pathophysiological interpretations across disease stages [10, 11]. The biomarker’s dual clinical implications further complicate threshold standardization. Elevated MFR correlates with improved survival in colon cancer [12, 13], yet emerging evidence suggests preserved visceral fat delays cachexia in advanced malignancies via IL-6/STAT3 pathway modulation [14]. This paradox underscores the urgency to develop adaptive thresholds accounting for both metabolic profiles and disease progression.

Current body composition assessment methods, such as bioelectrical impedance analysis, dual-energy X-ray absorptiometry, and conventional CT, have inherent limitations in accuracy and resolution [15]. Recent advancements in photon-counting CT (PCD-CT) provide a novel approach to noninvasive body composition analysis, allowing precise differentiation between muscle and fat tissues using energy-specific material decomposition techniques [16]. Despite previous studies exploring the prognostic impact of MFR in cancer, its specific role in diabetic gastric cancer patients remains underexplored.

This study aims to assess the prognostic significance of MFR quantified by PCD-CT in diabetic gastric cancer patients. By evaluating the relationship between MFR and postoperative outcomes, we seek to validate PCD-CT as a robust, noninvasive imaging tool for improving risk stratification and treatment planning.

## Methods

### Study design

This retrospective study was approved by the institutional Human Research Ethics Committee (Approval No: IR2024488) and waived the requirement for informed consent, adhering to the Declaration of Helsinki.

### Participants

**Fig. 1:**
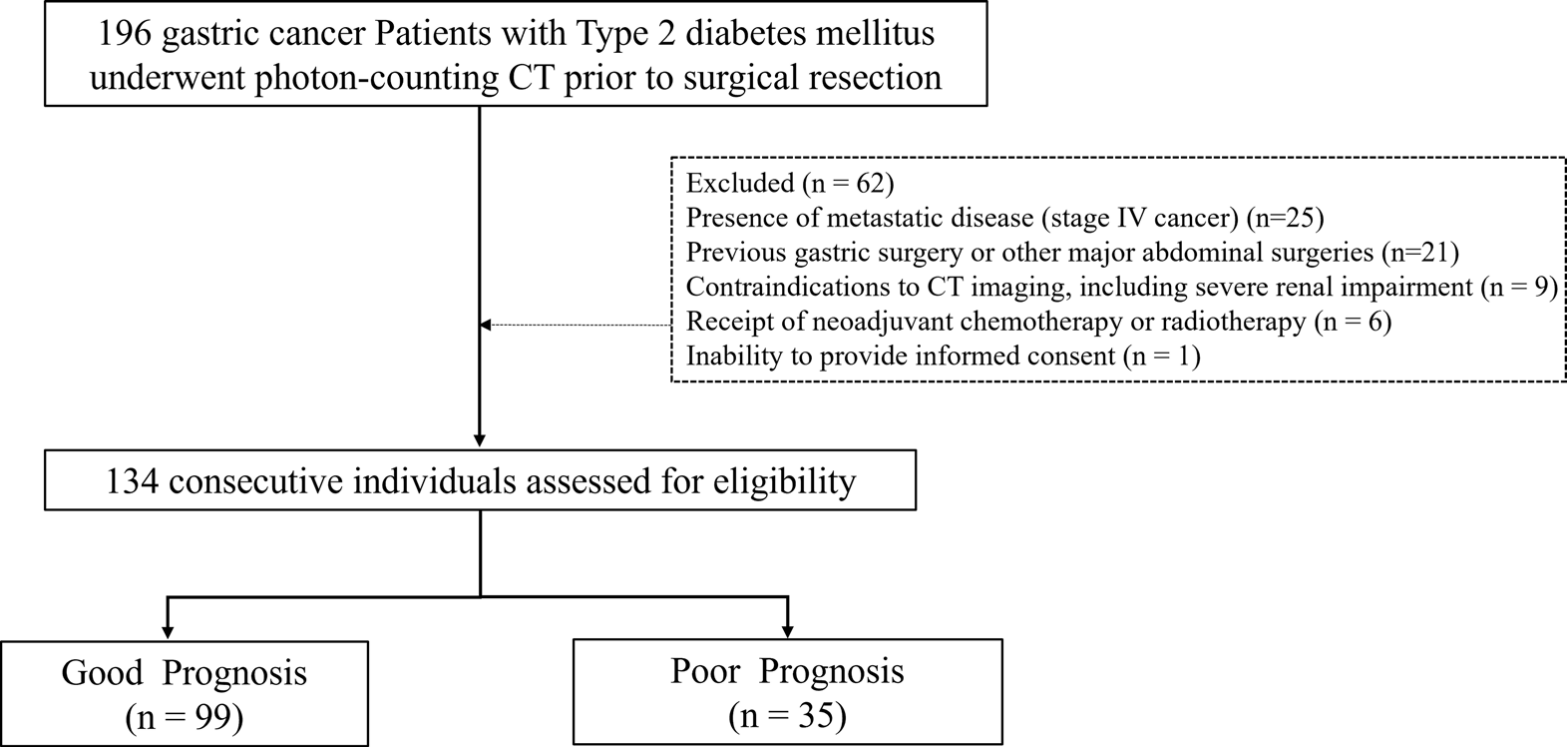
Flowchart of study participant eligibility

A total of 134 consecutive patients diagnosed with gastric cancer and type II diabetes mellitus were retrospectively analyzed. The study included patients who underwent elective surgical resection between August 2024 to December 2024. Inclusion Criteria: (1) Age ≥ 18 years; (2) Histologically confirmed gastric adenocarcinoma. (2) Diagnosis of type 2 diabetes mellitus [17]. (3) Scheduled for elective surgical resection of gastric cancer. (4) Preoperative PCD-CT scans performed within two weeks prior to surgery. Exclusion Criteria: (1) Presence of metastatic disease (Stage IV gastric cancer). (2) History of previous gastric or major abdominal surgery. (3) Contraindications to CT imaging (eGFR < 30 mL/min/1.73 m² or allergy to iodinated contrast media). (4) Receipt of neoadjuvant chemotherapy or radiotherapy. (5) Inability to provide informed consent. Preoperative clinical data, including age, sex, body mass index (BMI), Body Surface Area (BSA), comorbidities, cancer stage (TNM classification), were extracted from medical records. Figure 1 presents the subject flowchart for the study.

### CT examination

All scans were performed on a dual-source photon-counting CT system (NAEOTOM Alpha, Siemens Healthineers). Patients were instructed to fast for 12 h prior to scanning and received oral water for gastric distension, along with an anti-peristaltic agent to minimize motion artifacts. A triple-phase contrast-enhanced protocol was applied. Iodinated contrast material (350 mg I/mL) was administered at a dose of 1.5 mL/kg through the antecubital vein at a flow rate of 2.5–3.0 mL/s. Bolus tracking was used to initiate arterial phase scanning 25 s after reaching the threshold, followed by portal venous phase at 60 s and delayed phase at 120 s. Scanning parameters were as follows: 140 kV tube voltage, 144 × 0.4 mm collimation, 0.5-second gantry rotation time, pitch of 0.8, and tube current modulation (quality reference: 205 mAs). Axial images were reconstructed with a 1.0 mm slice thickness using a Qr40 kernel and iterative reconstruction at strength level 4.

### Imaging evaluation for clinical staging and body-composition measurements

Two independent observers interpreted the images every other day without knowing the clinical data. They relied on the assistance of enhanced CT for the assessment. In the initial staging of the primary tumor, the staging of tumor (T), lymph nodes (N), and metastasis (M), as well as the assessment of tumor size (longest diameter), were included [18].

**Fig. 2:**
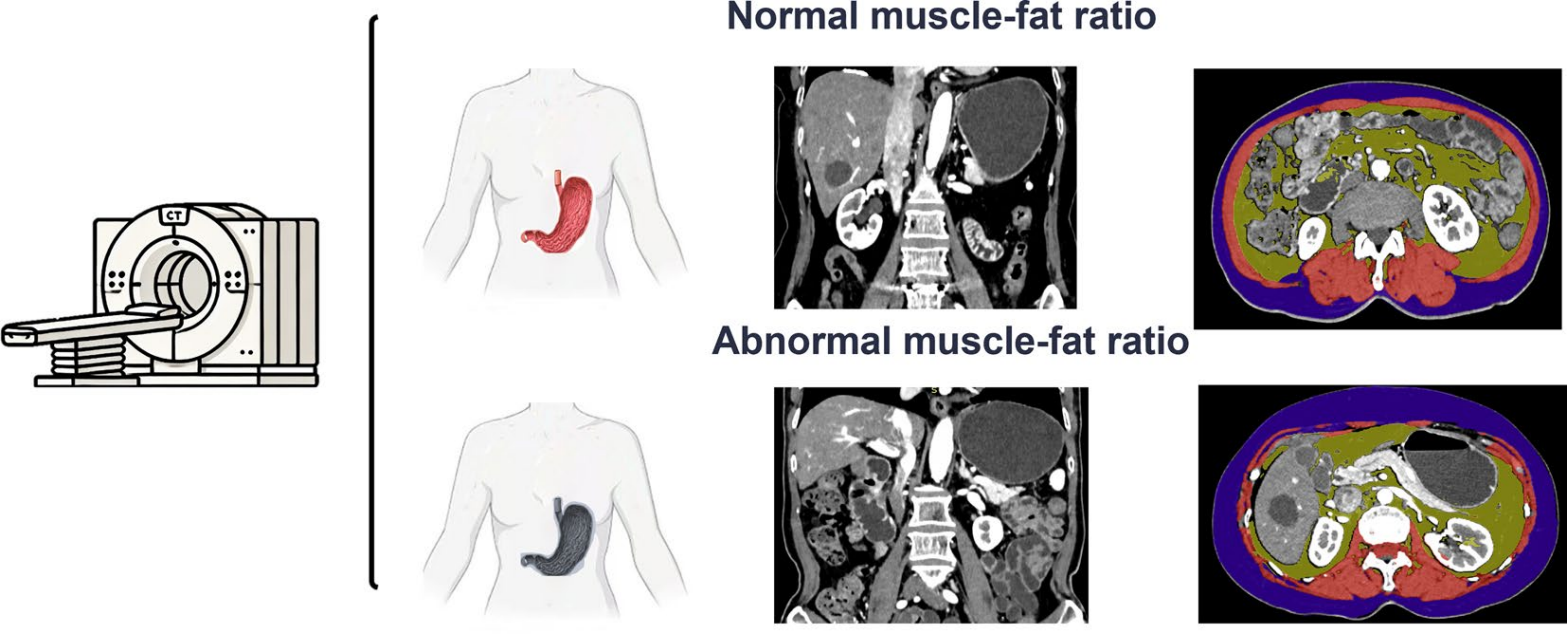
Body Composition measurement by photon-counting CT. Purple represents subcutaneous adipose tissue, yellow represents visceral adipose tissue, and red represents skeletal muscle

The body composition was analyzed by examining the images of the arterial phase, portal venous phase, delayed phase and non-enhanced phase, and the average values were obtained. Body composition, including subcutaneous adipose tissue (SAT), visceral adipose tissue (VAT), and Skeletal muscle (SM), was evaluated by semi-automated segmentation prototype (Cardiac Risk Assessment, syngo.via Frontier, Siemens Healthineers) and the CoreSlicer.com web-based software package (version 1.0.0; Montreal, Quebec, Canada). For abdominal SAT and VAT, CT images at the level of the third lumbar vertebra (L3) were analyzed, following established protocols [19]. SAT was defined as the fat between the skin and abdominal muscles, and VAT was defined as fat within the abdominal cavity, excluding SAT. The software automatically calculated these areas using a consistent HU threshold [20]. Skeletal muscle (psoas, paraspinal, and abdominal wall muscles) at L3 level was measured using a HU threshold range of -29 to 150, with the software calculating the cross-sectional area and mean attenuation, which reflect muscle density and overall health [21]. Measurements are shown in Fig. 2. To enhance the accuracy of metabolic parameter calculations, SAT, VAT, and muscle contents were adjusted for BMI and BSA.

### Outcome measures

Poor postoperative outcomes were defined based on existing literature as the occurrence of major postoperative complications classified as Clavien-Dindo grade III or higher [22]. These include complications requiring surgical, endoscopic, or radiological intervention (anastomotic leaks, postoperative bleeding, thromboembolic events) and surgical mortality [23].

### Statistical analysis

Statistical analysis was conducted using R version 4.1.2 (R Foundation for Statistical Computing). Continuous variables were compared using independent t-tests, while categorical variables were analyzed using chi-square tests. Group comparisons were performed using independent t-tests for continuous variables and chi-square (χ²) tests for categorical variables. Random survival forests are employed to identify muscle-fat ratio thresholds, with resampling techniques such as bootstrap validation rigorously assessing the stability of cutoff selection to ensure robustness in prognostic stratification. Logistic regression was also used to identify independent predictors of poor outcomes, with variables having *p* < 0.05 in the univariate analysis included in the multivariable analysis. Stepwise, backward selection was applied (*p* ≤ 0.05 for entry and *p* > 0.05 for removal). Three models were constructed for comparison: Model 1, adjusted for clinically significant covariates identified in multivariate analysis; Model 2, which included both the clinical model and body composition variables; and Model 3, which included both the clinical model and MFR. Receiver operating characteristic (ROC) curves were used to evaluate model performance, and the integrated discrimination improvement (IDI) and net reclassification improvement (NRI) were calculated to assess the improvement in predictive performance across the models. To evaluate the consistency of body composition measurements, data obtained from different observers and processed using different software platforms were compared using intraclass correlation coefficients (ICC) and Bland-Altman analysis, assessing both inter-observer and inter-software agreement.

## Results

### Patients’ characteristics

**Fig. 3:**
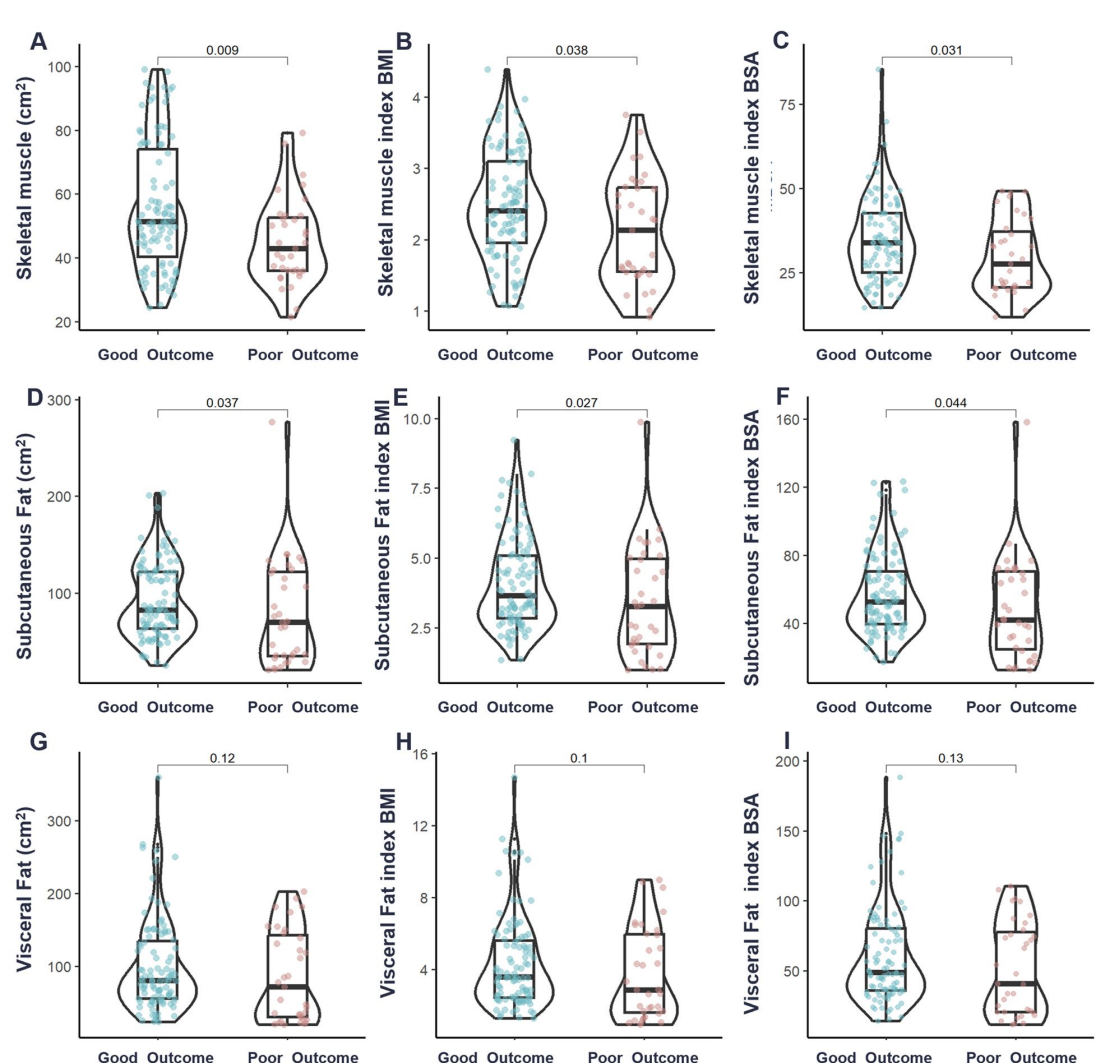
Group comparisons in different prognoses in body composition parameters are shown in (**A**) SM, (**B**) SM_BMI_, (**C**) SM_BSA_, (**D**) SF, (**E**) SF_BMI_, (**F**)SF_BSA_, (**G**)VF, (**H**) VF_BMI_ and (**I**) VF_BSA_. SM, Skeletal muscle; SM_BMI,_ Skeletal muscle index BMI; SM_BSA,_ Skeletal muscle index BSA; SF, Subcutaneous Fat; SF _BMI,_ Subcutaneous Fat index BMI; SF _BSA,_ Subcutaneous Fat index BSA; VF, Visceral Fat; VF _BMI,_ Visceral Fat index BMI; VF _BSA,_ Visceral Fat index BSA

A total of 134 patients were included in the study, with 99 patients classified as having a good outcome and 35 patients with a poor postoperative outcome. Across non-contrast, arterial, portal-venous, and delayed phases, visceral fat area, subcutaneous fat area, skeletal muscle area, and total fat area did not differ significantly (all *P* > 0.05; Table 1). The baseline clinical characteristics of the patients are presented in Table 2. Patients with a good outcomes had significantly higher skeletal muscle area (56.17 ± 19.86 cm²) compared to the poor outcomes group (45.47 ± 13.36 cm², *P* < 0.01). Similarly, the outcomes group exhibited higher SM_BMI_ (2.49 ± 0.75 vs. 2.14 ± 0.75, *P* = 0.02) and SM_BSA_ (35.11 ± 12.56 vs. 29.85 ± 11.06, *P* = 0.03) than the poor outcomes group (Fig. 3). The proportion of patients with abnormal MFR was significantly higher in the poor outcomes group (82.86% vs. 63.64%, *P* = 0.04). Other Baseline characteristics, showed no significant differences between groups.

**Table 1:** Comparison of body composition parameters across different CT phases

Variables	Average	Non-enhanced	Arterial Phase	Portal venous Phase	Delay Phase	*P*-value
	(*n* = 536)	(*n* = 134)	(*n* = 134)	(*n* = 134)	(*n* = 134)	
Visceral Fat area (cm^2^)	96.43 ± 62.68	96.56 ± 62.83	96.04 ± 62.86	96.58 ± 62.86	96.52 ± 62.88	1
Skeletal muscle area (cm^2^)	53.38 ± 18.88	53.48 ± 18.90	53.01 ± 18.95	53.50 ± 18.94	53.52 ± 18.96	1
Subcutaneous Fat area (cm^2^)	89.39 ± 43.88	89.52 ± 44.00	89.02 ± 44.01	89.50 ± 43.99	89.52 ± 44.01	1
Total fat (cm^2^)	185.41 ± 92.40	185.52 ± 92.65	185.06 ± 92.64	185.52 ± 92.66	185.54 ± 92.68	1

**Table 2:** Clinical characteristics at baseline

Variables	Total (*n* = 134)	Good Outcome (*n* = 99)	Poor Outcome	*P*-value
			(*n* = 35)	
Male, n (%)	78 (58.21)	57 (57.58)	21 (60.00)	0.8
Age in year	67.05 ± 10.60	66.87 ± 10.57	67.57 ± 10.84	0.74
High in cm	165.38 ± 7.65	165.10 ± 7.50	166.17 ± 8.10	0.48
Weigh in kg	61.83 ± 11.37	62.23 ± 12.16	60.69 ± 8.84	0.49
BMI in kg/ m^2^	22.45 ± 3.68	22.63 ± 3.78	21.93 ± 3.37	0.33
BSA in m^2^	1.61 ± 0.22	1.62 ± 0.21	1.59 ± 0.25	0.5
Alcohol, n (%)	63 (47.01)	42 (42.42)	21 (60.00)	0.07
Smoke, n (%)	66 (49.25)	51 (51.52)	15 (42.86)	0.38
Family history of gastric cancer, n (%)	13 (9.70)	11 (11.11)	2 (5.71)	0.55
Histology, n (%)				0.5
Moderate-differentiated	30 (22.39)	22 (22.22)	8 (22.86)	
Papillary	13 (9.70)	12 (12.12)	1 (2.86)	
Poor-differentiated	29 (21.64)	19 (19.19)	10 (28.57)	
Signet ring cell	19 (14.18)	14 (14.14)	5 (14.29)	
Well-differentiated	43 (32.09)	32 (32.32)	11 (31.43)	
TNM stage, n (%)				0.52
I	90 (67.16)	69 (69.70)	21 (60.00)	
II	24 (17.91)	17 (17.17)	7 (20.00)	
III	20 (14.93)	13 (13.13)	7 (20.00)	
T stage, n (%)				1
0	36 (26.87)	27 (27.27)	9 (25.71)	
I	41 (30.60)	30 (30.30)	11 (31.43)	
II	37 (27.61)	27 (27.27)	10 (28.57)	
III	20 (14.93)	15 (15.15)	5 (14.29)	
N stage, n (%)				0.34
0	32 (23.88)	24 (24.24)	8 (22.86)	
I	35 (26.12)	22 (22.22)	13 (37.14)	
II	44 (32.84)	34 (34.34)	10 (28.57)	
III	23 (17.16)	19 (19.19)	4 (11.43)	
Location, n (%)				0.33
Cardia	31 (23.13)	22 (22.22)	9 (25.71)	
Stomach antrum	43 (32.09)	29 (29.29)	14 (40.00)	
Stomach body	60 (44.78)	48 (48.48)	12 (34.29)	
ASA score, n (%)				0.08
1	47 (35.07)	39 (39.39)	8 (22.86)	
2	48 (35.82)	36 (36.36)	12 (34.29)	
3	39 (29.10)	24 (24.24)	15 (42.86)	
ECOG score, n (%)				0.69
0	44 (32.84)	33 (33.33)	11 (31.43)	
1	44 (32.84)	34 (34.34)	10 (28.57)	
2	46 (34.33)	32 (32.32)	14 (40.00)	
SM in cm^2^	53.38 ± 18.94	56.17 ± 19.86	45.47 ± 13.36	< 0.01
SM_BMI_	2.40 ± 0.76	2.49 ± 0.75	2.14 ± 0.75	0.02
SM_BSA_	33.74 ± 12.37	35.11 ± 12.56	29.85 ± 11.06	0.03
SF in cm^2^	89.39 ± 44.00	92.95 ± 39.17	79.32 ± 54.87	0.18
SF_BMI_	3.92 ± 1.74	4.10 ± 1.62	3.43 ± 1.97	0.04
SF_BSA_	55.17 ± 25.57	57.51 ± 23.62	48.54 ± 29.81	0.07
VF in cm^2^	96.43 ± 62.86	100.09 ± 63.14	86.06 ± 61.77	0.26
VF_BMI_	4.19 ± 2.52	4.34 ± 2.51	3.75 ± 2.52	0.23
VF_BSA_	58.36 ± 34.21	60.67 ± 34.49	51.84 ± 33.03	0.19
Total fat in cm^2^	185.41 ± 92.66	192.63 ± 86.60	165.00 ± 106.72	0.17
Abnormal MFR	92 (68.66)	63 (63.64)	29 (82.86)	**0.04**

Abbreviations: BMI, body mass index; ASA score, American Society of Anesthesiologists physical status classification; ECOG score, Eastern Cooperative Oncology Group Score; SM, Skeletal muscle ; SM_BMI,_ Skeletal muscle index BMI; SM_BSA,_ Skeletal muscle index BSA; SF, Subcutaneous Fat; SF _BMI,_ Subcutaneous Fat index BMI; SF _BSA,_ Subcutaneous Fat index BSA; VF, Visceral Fat; VF _BMI,_ Visceral Fat index BMI; VF _BSA,_ Visceral Fat index BSA; MFR, Muscle-to‐fat ratio

### Baseline characteristics according to MFR

**Fig. 4:**
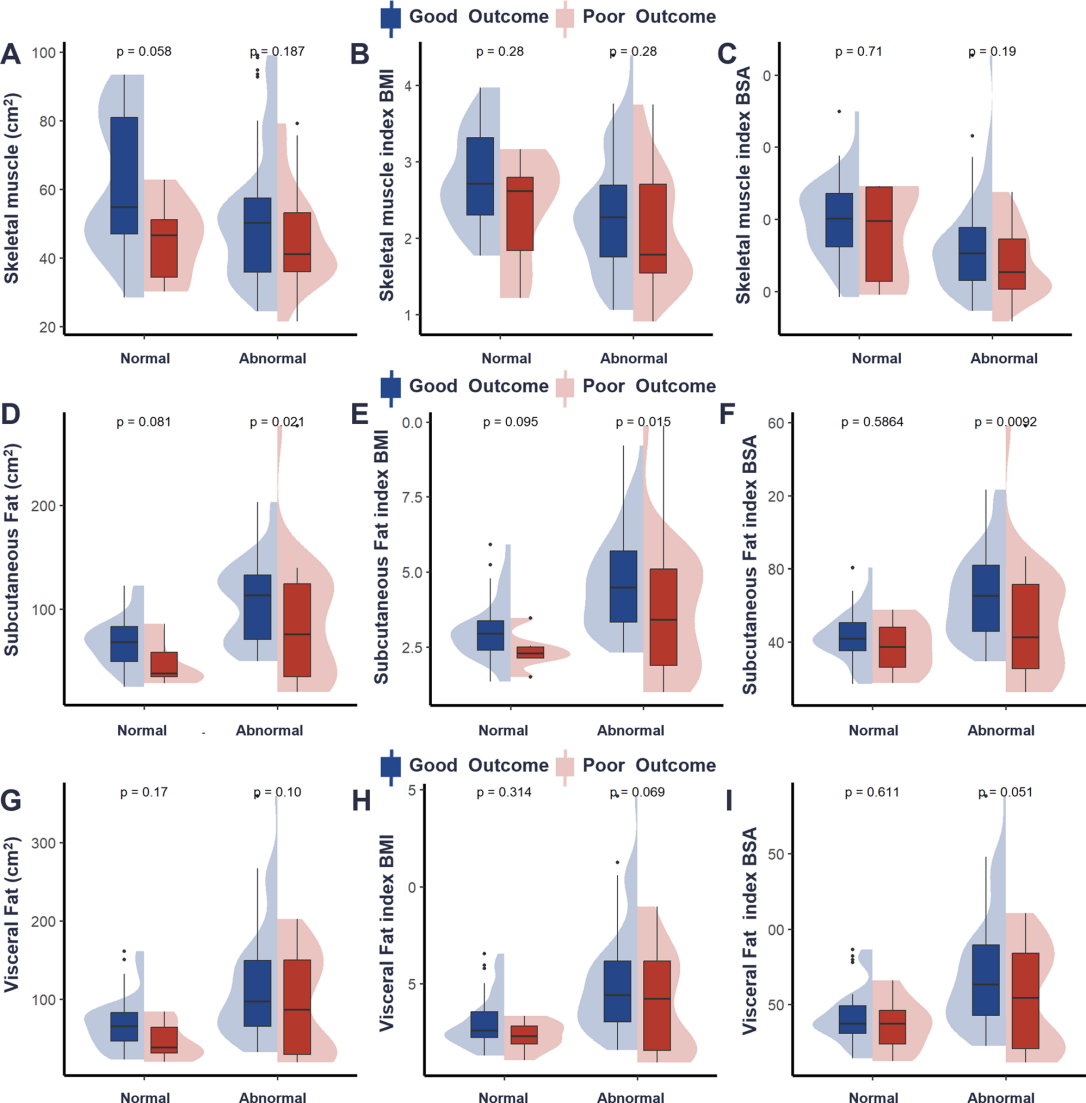
Group comparisons in Different prognoses between the normal and abnormal muscle-fat ratio with body composition parameters are shown in (**A**) SM, (**B**) SM_BMI_, (**C**) SM_BSA_, (**D**) SF, (**E**) SF_BMI_, (**F**)SF_BSA_, (**G**)VF, (**H**) VF_BMI_ and (**I**) VF_BSA_. SM, Skeletal muscle ; SM_BMI,_ Skeletal muscle index BMI; SM_BSA,_ Skeletal muscle index BSA; SF, Subcutaneous Fat; SF _BMI,_ Subcutaneous Fat index BMI; SF _BSA,_ Subcutaneous Fat index BSA; VF, Visceral Fat; VF _BMI,_ Visceral Fat index BMI; VF _BSA,_ Visceral Fat index BSA; MFR, Muscle-to‐fat ratio

Patients were categorized into a normal MFR group (MFR between 0.34 and 0.57, *n* = 42) and an abnormal MFR group (MFR < 0.34 or > 0.57, *n* = 92) based on the random forest analysis. Figure 4; Table 3 compares the baseline characteristics of patients categorized by normal (*n* = 42) and abnormal MFR (*n* = 92). Patients with abnormal MFR had a higher proportion of poor-differentiated histology (26.09% vs. 11.90%, *P* = 0.02). Additionally, patients with abnormal MFR had significantly lower skeletal muscle area (49.76 ± 17.65 cm² vs. 60.11 ± 19.96 cm², *P* < 0.01), SM_BMI_ (2.22 ± 0.76 vs. 2.72 ± 0.65, *P* < 0.01), and SM_BSA_ (30.97 ± 11.92 vs. 39.05 ± 11.59, *P* < 0.01). In contrast, subcutaneous fat area, SF_BMI_, and visceral fat area were significantly higher in the abnormal MFR group (*P* < 0.01 for all), indicating greater fat accumulation. Furthermore, the total fat area was significantly larger in the abnormal MFR group (222.95 ± 120.79 cm² vs. 127.60 ± 52.00 cm², *P* < 0.01).

**Table 3:** Baseline characteristics according to MFR

Variables	Total (*n* = 134)	Normal (*n* = 42)	Abnormal (*n* = 92)	*P*-value
Male, n (%)	78 (58.21)	29 (69.05)	49 (53.26)	0.09
Age in year)	67.05 ± 10.60	67.07 ± 10.16	67.04 ± 10.85	0.99
High in cm	165.38 ± 7.65	167.05 ± 7.66	164.62 ± 7.56	0.09
Weigh in kg	61.83 ± 11.37	61.12 ± 11.68	62.15 ± 11.28	0.63
BMI in kg/ m^2^	22.45 ± 3.68	21.99 ± 4.52	22.66 ± 3.23	0.39
BSA in m^2^	1.61 ± 0.22	1.55 ± 0.25	1.63 ± 0.20	0.06
Alcohol, n (%)	63 (47.01)	17 (40.48)	46 (50.00)	0.31
Smoke, n (%)	68 (50.75)	20 (47.62)	48 (52.17)	0.63
Family history of gastric cancer, n (%)	13 (9.70)	5 (11.90)	8 (8.70)	0.79
Histology, n(%)				**0.02**
Moderate-differentiated	30 (22.39)	7 (16.67)	23 (25.00)	
Papillary	13 (9.70)	3 (7.14)	10 (10.87)	
Poor-differentiated	29 (21.64)	5 (11.90)	24 (26.09)	
Signet ring cell	19 (14.18)	5 (11.90)	14 (15.22)	
Well-differentiated	43 (32.09)	22 (52.38)	21 (22.83)	
TNM stage, n (%)				0.09
I	90 (67.16)	24 (57.14)	66 (71.74)	
II	24 (17.91)	12 (28.57)	12 (13.04)	
III	20 (14.93)	6 (14.29)	14 (15.22)	
T stage, n (%)				0.89
0	36 (26.87)	11 (26.19)	25 (27.17)	
I	41 (30.60)	13 (30.95)	28 (30.43)	
II	37 (27.61)	13 (30.95)	24 (26.09)	
III	20 (14.93)	5 (11.90)	15 (16.30)	
N stage, n(%)				0.32
0	32 (23.88)	9 (21.43)	23 (25.00)	
I	35 (26.12)	10 (23.81)	25 (27.17)	
II	44 (32.84)	12 (28.57)	32 (34.78)	
III	23 (17.16)	11 (26.19)	12 (13.04)	
Location, n (%)				0.71
Cardia	31 (23.13)	8 (19.05)	23 (25.00)	
Stomach antrum	43 (32.09)	15 (35.71)	28 (30.43)	
Stomach body	60 (44.78)	19 (45.24)	41 (44.57)	
ASA score, n (%)				0.55
1	47 (35.07)	12 (28.57)	35 (38.04)	
2	48 (35.82)	17 (40.48)	31 (33.70)	
3	39 (29.10)	13 (30.95)	26 (28.26)	
ECOG score, n (%)				0.64
0	44 (32.84)	15 (35.71)	29 (31.52)	
1	44 (32.84)	15 (35.71)	29 (31.52)	
2	46 (34.33)	12 (28.57)	34 (36.96)	
SM in cm^2^	53.01 ± 18.95	60.11 ± 19.96	49.76 ± 17.65	**< 0.01**
SM_BMI_	2.38 ± 0.76	2.72 ± 0.65	2.22 ± 0.76	**< 0.01**
SM_BSA_	33.50 ± 12.36	39.05 ± 11.59	30.97 ± 11.92	**< 0.01**
SF in cm^2^	89.02 ± 44.01	65.06 ± 24.81	99.96 ± 46.55	**< 0.01**
SF_BMI_	3.91 ± 1.74	2.95 ± 0.99	4.35 ± 1.83	**< 0.01**
SF_BSA_	54.93 ± 25.58	41.84 ± 13.85	60.91 ± 27.47	**< 0.01**
VF in cm^2^	96.04 ± 62.86	64.45 ± 32.65	110.46 ± 68.00	**< 0.01**
VF_BMI_	4.17 ± 2.52	2.89 ± 1.29	4.76 ± 2.72	**< 0.01**
VF_BSA_	58.12 ± 34.22	41.13 ± 18.19	65.88 ± 36.99	**< 0.01**
Total fat in cm^2^	193.07 ± 113.08	127.60 ± 52.00	222.95 ± 120.79	**< 0.01**
Negative outcome	35 (26.12)	6 (14.29)	29 (31.52)	**0.02**

Abbreviations: BMI, body mass index; ASA score, American Society of Anesthesiologists physical status classification; ECOG score, Eastern Cooperative Oncology Group Score; SM, Skeletal muscle ; SM_BMI,_ Skeletal muscle index BMI; SM_BSA,_ Skeletal muscle index BSA; SF, Subcutaneous Fat; SF _BMI,_ Subcutaneous Fat index BMI; SF _BSA,_ Subcutaneous Fat index BSA; VF, Visceral Fat; VF _BMI,_ Visceral Fat index BMI; VF _BSA,_ Visceral Fat index BSA; MFR, Muscle-to‐fat ratio

### Logistic regression analysis

Univariate logistic regression identified an ASA score > 2 as a significant predictor of negative outcomes (OR = 3.05, 95% CI: 1.12–8.26, *p* = 0.03). Conversely, higher total muscle mass (OR = 0.96, 95% CI: 0.94–0.99, *p* = 0.01), MBMI (OR = 0.53, 95% CI: 0.31–0.92, *p* = 0.03), MBSA (OR = 0.96, 95% CI: 0.93–0.99, *p* = 0.03), subcutaneous fat BMI (OR = 0.78, 95% CI: 0.61–1.00, *p* = 0.05), and MFR (OR = 0.92, 95% CI: 0.88–0.98, *p* < 0.001) were associated with decreased odds of negative outcomes. After adjusting for confounders, abnormal MFR remained a strong predictor of negative outcomes (OR = 1.94, 95% CI: 1.17–2.58, *p* < 0.01). Alcohol consumption (OR = 1.76, 95% CI: 1.06–2.94, *p* = 0.03) and an ASA score greater than 2 (OR = 2.91, 95% CI: 1.04–8.16, *p* = 0.04) were also significant predictors (Table 4).

**Table 4:** Univariable and multivariable logistic regression analysis for outcome

Variables	OR (95%CI)	*P*-value	OR (95%CI)	*P*-value
Sex, n (%)	1.11 (0.50 ~ 2.42)	0.80		
Age	1.01 (0.97 ~ 1.04)	0.74		
High	1.02 (0.97 ~ 1.07)	0.48		
Weigh	0.99 (0.95 ~ 1.02)	0.49		
BMI	0.95 (0.85 ~ 1.06)	0.33		
BSA	0.55 (0.10 ~ 3.08)	0.50		
Alcohol, n (%)	2.04 (0.93 ~ 4.46)	0.03	1.76(1.06–2.94)	0.03
Smoke, n (%)	1.42 (0.65 ~ 3.08)	0.38		
Family history of gastric cancer, n (%)	0.48 (0.10 ~ 2.30)	0.36		
Histology, n (%)				
Papillary	0.23 (0.03 ~ 2.06)	0.19		
Poor-differentiated	1.45 (0.48 ~ 4.41)	0.52		
Signet ring cell	0.98 (0.27 ~ 3.61)	0.98		
Well-differentiated	0.95 (0.33 ~ 2.73)	0.92		
TNM stage > II	1.77 (0.62 ~ 5.01)	0.28		
T stage > II	1.00 (0.28 ~ 3.53)	1.00		
N stage > II	0.63 (0.16 ~ 2.42)	0.50		
Location Stomach body	0.61 (0.22 ~ 1.66)	0.34		
ASA score > 2	3.05 (1.12 ~ 8.26)	0.03	2.91 (1.04 ~ 8.16)	0.04
ECOG score > 1	1.31 (0.52 ~ 3.32)	0.57		
SM in cm^2^	0.96 (0.94 ~ 0.99)	0.01	0.95 (0.83 ~ 1.09)	0.47
SM_BMI_	0.53 (0.31 ~ 0.92)	0.03	1.59 (0.35 ~ 7.29)	0.59
SM_BSA_	0.96 (0.93 ~ 0.99)	0.03	1.01 (0.92 ~ 1.10)	0.89
SF in cm^2^	1.00 (0.99 ~ 1.00)	0.26		
SF_BMI_	0.99 (0.98 ~ 1.00)	0.19		
SF_BSA_	0.90 (0.76 ~ 1.07)	0.23		
VF in cm^2^	0.99 (0.98 ~ 1.00)	0.12		
VF_BMI_	0.98 (0.97 ~ 1.00)	0.08		
VF_BSA_	0.78 (0.61 ~ 1.00)	0.03	0.76 (0.57 ~ 0.99)	0.06
Total fat in cm^2^	1.00 (1.00 ~ 1.00)	0.61		
MFR abnormal	0.92 (0.88 ~ 0.98)	< 0.01	1.94 (1.17 ~ 2.58)	< 0.01

Abbreviations: BMI, body mass index; ASA score, American Society of Anesthesiologists physical status classification; ECOG score, Eastern Cooperative Oncology Group Score; SM, Skeletal muscle ; SM_BMI,_ Skeletal muscle index BMI; SM_BSA,_ Skeletal muscle index BSA; SF, Subcutaneous Fat; SF _BMI,_ Subcutaneous Fat index BMI; SF _BSA,_ Subcutaneous Fat index BSA; VF, Visceral Fat; VF _BMI,_ Visceral Fat index BMI; VF _BSA,_ Visceral Fat index BSA; MFR, Muscle-to‐fat ratio

### Predictors of outcomes

The prediction model for outcomes is summarized in Table 5. Model 1, which included alcohol and ASA score, had an AUC of 0.66, sensitivity of 0.54, and specificity of 0.77. Model 2, incorporating skeletal muscle-related parameters, improved the performance slightly (AUC = 0.69, sensitivity = 0.60, specificity = 0.71). Model 3, which included alcohol, ASA score, and MFR, exhibited the best predictive performance, with an AUC of 0.75, sensitivity of 0.74, and specificity of 0.71. The NRI (0.52, *P* < 0.01) and IDI (0.09, *P* < 0.01) confirmed the model’s superior predictive accuracy.

**Table 5:** Prediction model for outcome

	NRI	*P* value	IDI	*P* value	AUC	SEN	SPE
Model 1	Reference	-	Reference	-	0.66	0.54	0.77
Model 2	0.38 [ 0.08–0.71]	0.05	0.03 [0.01–0.06]	0.02	0.69	0.60	0.71
Model 3	0.52 [ 0.16–0.88]	< 0.01	0.09 [ 0.04–0.13]	< 0.01	0.75	0.74	0.71

IDI, integrated discrimination improvement; NRI, net reclassification index

Model 1: Alcohol + ASA score

Model 2: Alcohol + ASA score + SM + SM_BMI_+SM_BSA_

Model 3: Alcohol + ASA score + MFR

Abbreviations: ASA score, American Society of Anesthesiologists physical status classification, SM, Skeletal muscle ; SM_BMI,_ Skeletal muscle index BMI; SM_BSA,_ Skeletal muscle index BSA, MFR, Muscle-to‐fat ratio

### Intra‑ and inter‑observer variability

The as detailed in the supplementary materials table demonstrates high consistency between different observers and software, with intraclass correlation coefficients (ICC) above 0.90 and narrow limits of agreement in the Bland-Altman analysis, indicating excellent reproducibility in body composition measurements.

## Discussion

This study investigated the prognostic value of photon-counting CT (PCCT)–derived muscle-fat ratio (MFR) in diabetic gastric cancer patients undergoing curative surgery. We found that higher MFR, reflecting a greater proportion of skeletal muscle relative to adipose tissue, was independently associated with better postoperative outcomes. Notably, CT attenuation values for muscle and fat were consistent across non-contrast, arterial, portal venous, and delayed phases, allowing us to average measurements across phases. This stability underscores the robustness of PCCT for body-composition analysis and supports our strategy of calculating MFR from multi-phase data. Overall, our results highlight MFR as a novel imaging biomarker that improves risk stratification beyond clinical factors alone.

Body composition has emerged as a critical determinant of cancer outcomes, encapsulating metabolic health, nutritional status, and the ability to withstand the physiological stress of surgery [24]. Traditional metrics like BMI are inadequate for capturing these complexities, as they do not distinguish between muscle and fat distribution [25]. In contrast, MFR integrates both muscle mass and fat accumulation, offering a more precise measure of a patient’s metabolic health and surgical resilience [26]. In patients with metabolic comorbidities such as diabetes and obesity, concomitant muscle wasting and visceral adiposity further complicate risk stratification [27]. This imbalance complicates both nutritional assessment and postoperative risk prediction. Our study emphasizes the role of MFR in predicting surgical complications, particularly in diabetic gastric cancer patients, where the interplay between sarcopenia and excess visceral fat is further exacerbated by metabolic dysfunction.

In our cohort of diabetic gastric cancer patients, a lower preoperative MFR was strongly associated with postoperative complications such as infections, delayed wound healing, and extended hospital stays, highlighting the need for a more integrated approach to body composition evaluation. This is consistent with previous studies demonstrating that reduced muscle mass correlates with increased risks of infections, delayed wound healing, and prolonged hospital stays [28]. A meta-analysis showed that sarcopenia in gastric cancer patients was associated with an increased risk of infections and delayed recovery [29]. By capturing both muscle depletion and fat excess, MFR offers a more nuanced framework for predicting adverse outcomes, particularly in the sarcopenic-obesity phenotype, a recognized independent predictor of postoperative morbidity [30]. Notably, we also observed a significant difference in histological grade between MFR groups: patients with abnormal MFR had a greater proportion of poorly differentiated tumors and fewer well‐differentiated lesions. Poorly differentiated gastric cancers are inherently more aggressive, exhibiting rapid proliferation and heightened systemic inflammation, which can accelerate muscle catabolism and alter fat distribution. This bidirectional interplay suggests that an unfavorable MFR not only reflects compromised host reserve but also correlates with underlying tumor biology. Importantly, in multivariable models adjusting for histological grade, MFR remained an independent predictor of poor outcome, highlighting its value beyond traditional staging parameters.

In abdominal CT imaging, the lack of standardization and appropriate quality control in contrast-enhanced phases poses a significant challenge, particularly in the analysis of body composition in patients with abdominal-related diseases [31]. Most prognostic studies rely on the venous phase of CT, as it has been shown that lower skeletal muscle mass or higher visceral adiposity can predict poorer outcomes, including survival and postoperative complications [32, 33]. However, some studies have compared the use of different CT phases (plain, arterial, and venous), for muscle and fat measurement, finding that muscle area remains relatively stable across phases, while muscle density and fat indices can vary significantly [34, 35]. This variability can introduce phase-specific biases, particularly due to fluctuations in muscle Hounsfield Units (HU) during arterial enhancement. To address this issue, our study employed a multi-phase averaging approach, incorporating non-enhanced, arterial, portal venous, and delayed phases. While the portal venous phase is commonly used for body composition analysis, there is no consensus on its superiority. By averaging across all phases, we aim to reduce stochastic noise and improve measurement stability, similar to signal-averaging techniques validated in quantitative imaging. Studies have shown that automated body composition tools exhibit high reproducibility across various CT protocols, reinforcing the value of multi-phase averaging in enhancing the robustness and generalizability of body composition metrics [36].

Interestingly, our results found no statistical differences in body composition metrics across the four CT phases when using PCD-CT. This finding suggests that accurate measurements can be obtained from a single CT phase, such as the non-enhanced scan, which can be particularly advantageous in patients at risk for contrast-induced nephropathy or those with contrast allergies. The promising potential of PCD-CT in body composition analysis is further supported by studies demonstrating its high accuracy and reproducibility in fat quantification, even across varying radiation doses and reconstruction methods [37]. A recent phantom study showed that PCD-CT fat-fraction measurements were highly accurate and stable across different radiation doses and reconstruction protocols, reinforcing its reliability in fat quantification [38]. Both phantom and early clinical studies have demonstrated that PCD-CT can accurately and reproducibly quantify adipose tissue across a range of imaging conditions. For example, PCD-CT at a 70 keV virtual monochromatic setting achieved fat quantification with excellent agreement to reference values, producing epicardial fat attenuation values nearly identical to conventional CT [39]. Moreover, PCD-CT offers superior spatial resolution and reduced electronic noise compared to conventional CT, enhancing the precision of adipose tissue quantification [40, 41]. These capabilities highlight the potential of PCD-CT to significantly improve prognostic assessments, particularly in preoperative body composition analysis for patients with diabetes and gastric cancer.

Although this study provides promising findings, several limitations should be considered. First, the sample size is relatively small, and potential confounding factors, such as inflammatory conditions, should be controlled to improve the generalizability of the results. While the muscle-fat ratio (MFR) shows strong predictive potential, it does not account for muscle function or overall physical fitness, which are also critical determinants of patient outcomes. Future studies should incorporate functional evaluations to enhance the predictive value of MFR. Additionally, although our analysis focused on diabetic gastric cancer patients, the applicability of MFR in non-diabetic populations requires validation. Further investigations are needed to explore the broader use of MFR in different oncological contexts. Despite finding consistent body composition measurements across various CT scanning phases, multi-center validation studies are essential to confirm the robustness and reproducibility of these results, especially for non-enhanced scans. Finally, while we observed high consistency between two post-processing software platforms, future research should investigate the integration of open-source tools and AI-driven segmentation techniques to improve automation and clinical workflow, ultimately enhancing the practical application of these methods in routine oncology practice.

## Conclusion

This study highlights the potential of PCD-CT-derived MFR as a reliable and accurate prognostic biomarker in diabetic gastric cancer patients. MFR demonstrates strong predictive value for postoperative complications and survival outcomes, reinforcing its role in surgical risk stratification. PCD-CT, with its high precision and noninvasive nature, offers a powerful tool for quantitative body composition analysis.

## Supplementary Information

Below is the link to the electronic supplementary material.

Supplementary Material 1

## Data Availability

The data analyzed and presented in this study are available from the corresponding author upon reasonable request.
