# Supplementary material for: Enhancing risk stratification in diabetic gastric cancer: muscle-fat ratio from photon-counting CT as a predictor of postoperative complications
Source: BMC Med Imaging. 2025 Aug 19;25:334. doi: 10.1186/s12880-025-01872-1 (PMC12363078; doi:10.1186/s12880-025-01872-1)
Supplement: Supplementary file 1 — Supplementary Material 1 [file 12880_2025_1872_MOESM1_ESM.docx]

Supplementary materials Table : Consistency of Body Composition Measurements Across Different Observers and Software Platforms, as Assessed by Intraclass Correlation Coefficients (ICC) and Bland-Altman Analysis

| **Comparison Group** | **Intraclass Correlation Coefficient (ICC)** | **95% Confidence Interval (CI)** | **Bland-Altman Mean Difference** | **Bland-Altman Limits of Agreement (LoA)** |
| --- | --- | --- | --- | --- |
| **Observer** | | | | |
| Non-enhanced Visceral Fat area (cm2) | 0.94 | (0.88, 0.98) | 0.08 | (-0.15, 0.31) |
| Arterial Phase Visceral Fat area (cm2) | 0.92 | (0.85, 0.97) | 0.12 | (-0.1, 0.34) |
| Portal venous Phase Visceral Fat area (cm2) | 0.92 | (0.86, 0.97) | 0.04 | (-0.09, 0.17) |
| Delay Phase Visceral Fat area (cm2) | 0.91 | (0.84, 0.96) | 0.09 | (-0.12, 0.3) |
| Non-enhanced Subcutaneous Fat area (cm2) | 0.91 | (0.84, 0.96) | 0.08 | (-0.10, 0.26) |
| Arterial Phase Subcutaneous Fat area (cm2) | 0.93 | (0.87, 0.97) | -0.02 | (-0.12, 0.08) |
| Portal venous Phase Subcutaneous Fat area (cm2) | 0.94 | (0.88, 0.98) | 0.06 | (-0.07, 0.19) |
| Delay Phase Subcutaneous Fat area (cm2) | 0.94 | (0.89, 0.98) | 0.07 | (-0.06, 0.20) |
| Non-enhanced Skeletal muscle area (cm2) | 0.95 | (0.90, 0.98) | 0.1 | (-0.2, 0.4) |
| Arterial Phase Skeletal muscle area (cm2) | 0.94 | (0.89, 0.98) | 0.07 | (-0.08, 0.22) |
| Portal venous Phase Skeletal muscle area (cm2) | 0.95 | (0.90, 0.98) | 0.05 | (-0.10, 0.20) |
| Delay Phase Skeletal muscle area (cm2) | 0.92 | (0.85, 0.97) | 0.09 | (-0.08, 0.26) |
| **Software** | | | | |
| Non-enhanced Visceral Fat area (cm2) | 0.98 | (0.94, 0.99) | 0.06 | (-0.05, 0.17) |
| Arterial Phase Visceral Fat area (cm2) | 0.97 | (0.93, 0.99) | -0.01 | (-0.08, 0.06) |
| Portal venous Phase Visceral Fat area (cm2) | 0.96 | (0.91, 0.98) | -0.05 | (-0.2, 0.1) |
| Delay Phase Visceral Fat area (cm2) | 0.97 | (0.92, 0.99) | 0.04 | (-0.08, 0.16) |
| Non-enhanced Subcutaneous Fat area (cm2) | 0.96 | (0.91, 0.98) | -0.03 | (-0.15, 0.09) |
| Arterial Phase Subcutaneous Fat area (cm2) | 0.96 | (0.90, 0.98) | 0.1 | (-0.07, 0.27) |
| Portal venous Phase Subcutaneous Fat area (cm2) | 0.97 | (0.92, 0.99) | 0.02 | (-0.08, 0.12) |
| Delay Phase Subcutaneous Fat area (cm2) | 0.98 | (0.94, 0.99) | 0.04 | (-0.05, 0.13) |
| Non-enhanced Skeletal muscle area (cm2) | 0.99 | (0.95, 0.99) | -0.01 | (-0.09, 0.07) |
| Arterial Phase Skeletal muscle area (cm2) | 0.96 | (0.90, 0.98) | 0.06 | (-0.07, 0.19) |
| Portal venous Phase Skeletal muscle area (cm2) | 0.97 | (0.93, 0.99) | 0.03 | (-0.06, 0.12) |
| Delay Phase Skeletal muscle area (cm2) | 0.98 | (0.94, 0.99) | -0.02 | (-0.10, 0.06) |
